# 2-Phenyl-1-(phenyl­sulfin­yl)naphtho[2,1-*b*]furan

**DOI:** 10.1107/S1600536809019977

**Published:** 2009-05-29

**Authors:** Hong Dae Choi, Pil Ja Seo, Byeng Wha Son, Uk Lee

**Affiliations:** a Department of Chemistry, Dongeui University, San 24 Kaya-dong Busanjin-gu, Busan 614-714, Republic of Korea; b Department of Chemistry Pukyong National University 599-1 Daeyeon 3-dong, Nam-gu, Busan 608-737, Republic of Korea

## Abstract

In the title compound, C_24_H_16_O_2_S, the O atom and the phenyl group of the phenyl­sulfinyl substituent lie on opposite sides of the plane of the naphthofuran fragment; the phenyl ring is almost perpendicular to this plane [82.34 (5)°]. The 2-phenyl ring is rotated out of the naphthofuran plane making a dihedral angle of 48.21 (6)°. The crystal structure shows π–π inter­actions between the central benzene rings of adjacent mol­ecules [centroid–centroid distance = 3.516 (3) Å], as well as non-classical C—H⋯O hydrogen bonds.

## Related literature

For the crystal structures of similar naphtho[2,1-*b*]furan derivatives, see: Choi *et al.* (2007 ▶, 2008 ▶). For the biological and pharmacological activity of naphthofuran compounds, see: Goel & Dixit (2004 ▶); Hagiwara *et al.* (1999 ▶); Piloto *et al.* (2005 ▶).
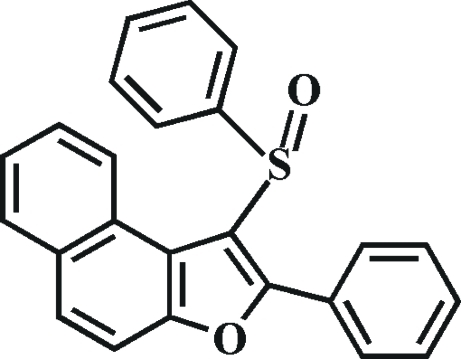


## Experimental

### 

#### Crystal data

C_24_H_16_O_2_S
*M* *_r_* = 368.43
Triclinic, 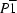

*a* = 9.2262 (7) Å
*b* = 10.3430 (8) Å
*c* = 10.4296 (8) Å
α = 78.298 (1)°
β = 86.849 (1)°
γ = 67.506 (1)°
*V* = 900.15 (12) Å^3^
*Z* = 2
Mo *K*α radiation
μ = 0.20 mm^−1^
*T* = 173 K
0.30 × 0.20 × 0.10 mm

#### Data collection

Bruker SMART CCD diffractometer
Absorption correction: none
6736 measured reflections
3140 independent reflections
2602 reflections with *I* > 2σ(*I*)
*R*_int_ = 0.076

#### Refinement

*R*[*F* ^2^ > 2σ(*F* ^2^)] = 0.040
*wR*(*F* ^2^) = 0.106
*S* = 1.06
3140 reflections
244 parameters
H-atom parameters constrained
Δρ_max_ = 0.30 e Å^−3^
Δρ_min_ = −0.36 e Å^−3^

### 

Data collection: *SMART* (Bruker, 2001 ▶); cell refinement: *SAINT* (Bruker, 2001 ▶); data reduction: *SAINT*; program(s) used to solve structure: *SHELXS97* (Sheldrick, 2008 ▶); program(s) used to refine structure: *SHELXL97* (Sheldrick, 2008 ▶); molecular graphics: *ORTEP-3* (Farrugia, 1997 ▶) and *DIAMOND* (Brandenburg, 1998 ▶); software used to prepare material for publication: *SHELXL97*.

## Supplementary Material

Crystal structure: contains datablocks global, I. DOI: 10.1107/S1600536809019977/ng2587sup1.cif

Structure factors: contains datablocks I. DOI: 10.1107/S1600536809019977/ng2587Isup2.hkl

Additional supplementary materials:  crystallographic information; 3D view; checkCIF report

## Figures and Tables

**Table 1:** Hydrogen-bond geometry (Å, °)

*D*—H⋯*A*	*D*—H	H⋯*A*	*D*⋯*A*	*D*—H⋯*A*
C9—H9⋯O2^i^	0.95	2.59	3.488 (3)	158
C18—H18⋯O2^ii^	0.95	2.57	3.323 (3)	137

Symmetry codes: (i) 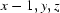; (ii) 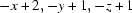.

**Fig. 1.**
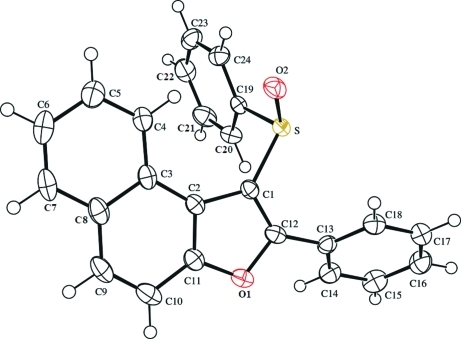
The molecular structure of the title compound with the atom numbering scheme. Displacement ellipsoids are drawn at the 50% probability level. H atoms are presented as a small spheres of arbitrary radius.

**Fig. 2.**
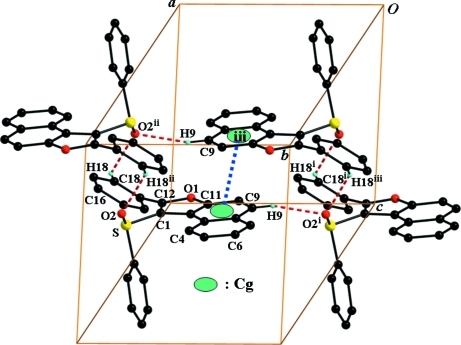
The π–π and C–H···O interactions (dotted lines) in the title compound. Cg denotes the ring centroids. [Symmetry code: (i) x - 1, y, z; (ii) -x + 2, -y + 1, -z + 1; (iii) -x + 1, -y + 1, -z + 1.]

## supplementary crystallographic information

### Comment

The naphthofuran ring system has attracted widespread interest in view of their biological and pharmacological activities (Goel & Dixit, 2004; Hagiwara *et al.*, 1999; Piloto *et al.*, 2005). This work is related to our communications on the synthesis and structures of naphtho[2,1-b]furan analogues, *viz*. 2-methyl-1-(phenylsulfinyl)naphtho[2,1-b]furan (Choi *et al.*, 2007) and 2-methyl-1-(phenylsulfonyl)naphtho[2,1-b]furan (Choi *et al.*, 2008). We present the crystal structure of the title compound (I), 2-phenyl-1-(phenylsulfinyl)naphtho[2,1-b]furan (Fig. 1).

The naphthofuran unit is essentially planar, with a mean deviation of 0.015 (2) Å from the least-squares plane defined by the thirteen constituent atoms. The dihedral angle in (I) formed by the plane of the naphthofuran ring and the plane of 2-phenyl ring is 48.21 (6)°, and the phenyl ring (C19-C24) with 82.34 (5)° lies toward the naphthofuran plane. The crystal packing (Fig. 2) is stabilized by aromatic π–π interactions between the central benzene rings from the adjacent molecules. The Cg···Cg^iii^ distance is 3.516 (3) Å (Cg is the centroide of the C2/C3/C8/C9/C10/C11 benzene ring, symmetry code as in Fig. 2). The molecular packing is further stabilized by weak non-classical intermolecular C–H···O hydrogen bonds, the first between an aromatic H atom of the naphthofuran fragment and the S═O unit, the second between an aromatic H atom of 2-phenyl ring and the S═O unit, respectively (Table 1 and Fig. 2; symmetry code as in Fig. 2).

### Experimental

The 77% 3-chloroperoxybenzoic acid (77%, 247 mg, 1.1 mmol) was added in small portions to a stirred solution of 2-phenyl-1-(phenylsulfanyl)naphtho[2,1-b]furan (352 mg, 1.0 mmol) in dichloromethane (30 mL) at 273 K. After being stirred at room temperature for 3h, the mixture was washed with saturated sodium bicarbonate solution and the organic layer was separated, dried over magnesium sulfate, filtered and concentrated in vacuum. The residue was purified by column chromatography (hexane-ethyl acetate, 2:1 v/v) to afford the title compound as a colorless solid [yield 81%, m.p. 462-463 K; R_f_ = 0.54 (hexane-ethyl acetate, 2:1 v/v)]. Single crystals suitable for X-ray diffraction were prepared by slow evaporation of a solution of the title χompound in benzene at room temperature.

### Refinement

All H atoms were positioned geometrically and refined using a riding model, with C–H = 0.95 Å for aromatic H atoms and with Uiso(H) = 1.2Ueq (C) for aromatic H atoms.

### Crystal data

**Table:**

C_24_H_16_O_2_S	*Z* = 2
*M**_r_* = 368.43	*F*(000) = 384
Triclinic, *P*1	*D*_x_ = 1.359 Mg m^−^^3^
Hall symbol: -P 1	Mo *K*α radiation, λ = 0.71073 Å
*a* = 9.2262 (7) Å	Cell parameters from 3903 reflections
*b* = 10.3430 (8) Å	θ = 2.2–28.1°
*c* = 10.4296 (8) Å	µ = 0.20 mm^−^^1^
α = 78.298 (1)°	*T* = 173 K
β = 86.849 (1)°	Block, colorless
γ = 67.506 (1)°	0.30 × 0.20 × 0.10 mm
*V* = 900.15 (12) Å^3^	

### Data collection

**Table:**

Bruker SMART CCD diffractometer	2602 reflections with *I* > 2σ(*I*)
Radiation source: fine-focus sealed tube	*R*_int_ = 0.076
graphite	θ_max_ = 25.0°, θ_min_ = 2.6°
Detector resolution: 10.0 pixels mm^-1^	*h* = −10→10
φ and ω scans	*k* = −12→12
6736 measured reflections	*l* = −12→12
3140 independent reflections	

### Refinement

**Table:**

Refinement on *F*^2^	Primary atom site location: structure-invariant direct methods
Least-squares matrix: full	Secondary atom site location: difference Fourier map
*R*[*F*^2^ > 2σ(*F*^2^)] = 0.040	Hydrogen site location: difference Fourier map
*wR*(*F*^2^) = 0.106	H-atom parameters constrained
*S* = 1.06	*w* = 1/[σ^2^(*F*_o_^2^) + (0.0292*P*)^2^ + 0.4516*P*] where *P* = (*F*_o_^2^ + 2*F*_c_^2^)/3
3140 reflections	(Δ/σ)_max_ < 0.001
244 parameters	Δρ_max_ = 0.30 e Å^−^^3^
0 restraints	Δρ_min_ = −0.36 e Å^−^^3^

### Special details

**Table:**

Geometry. All esds (except the esd in the dihedral angle between two l.s. planes) are estimated using the full covariance matrix. The cell esds are taken into account individually in the estimation of esds in distances, angles and torsion angles; correlations between esds in cell parameters are only used when they are defined by crystal symmetry. An approximate (isotropic) treatment of cell esds is used for estimating esds involving l.s. planes.
Refinement. Refinement of F^2^ against ALL reflections. The weighted R-factor wR and goodness of fit S are based on F^2^, conventional R-factors R are based on F, with F set to zero for negative F^2^. The threshold expression of F^2^ > 2sigma(F^2^) is used only for calculating R-factors(gt) etc. and is not relevant to the choice of reflections for refinement. R-factors based on F^2^ are statistically about twice as large as those based on F, and R-factors based on ALL data will be even larger.

### Fractional atomic coordinates and isotropic or equivalent isotropic displacement parameters (Å^2^)

**Table:**

	*x*	*y*	*z*	*U*_iso_*/*U*_eq_	
S	0.92526 (5)	0.67298 (6)	0.63521 (5)	0.02249 (16)	
O1	0.55628 (15)	0.80588 (16)	0.40427 (15)	0.0287 (4)	
O2	1.01571 (15)	0.51840 (16)	0.68794 (15)	0.0304 (4)	
C1	0.7484 (2)	0.6976 (2)	0.5575 (2)	0.0217 (4)	
C2	0.6230 (2)	0.6466 (2)	0.5980 (2)	0.0225 (5)	
C3	0.5963 (2)	0.5480 (2)	0.7043 (2)	0.0247 (5)	
C4	0.7034 (2)	0.4667 (2)	0.8087 (2)	0.0288 (5)	
H4	0.8013	0.4767	0.8114	0.035*	
C5	0.6685 (3)	0.3735 (3)	0.9063 (2)	0.0369 (6)	
H5	0.7423	0.3195	0.9760	0.044*	
C6	0.5244 (3)	0.3568 (3)	0.9045 (3)	0.0409 (6)	
H6	0.5006	0.2928	0.9733	0.049*	
C7	0.4196 (3)	0.4323 (3)	0.8042 (3)	0.0367 (6)	
H7	0.3231	0.4194	0.8033	0.044*	
C8	0.4506 (2)	0.5296 (2)	0.7011 (2)	0.0304 (5)	
C9	0.3413 (2)	0.6054 (3)	0.5950 (3)	0.0350 (6)	
H9	0.2462	0.5900	0.5946	0.042*	
C10	0.3676 (2)	0.6990 (3)	0.4943 (2)	0.0341 (6)	
H10	0.2936	0.7499	0.4242	0.041*	
C11	0.5109 (2)	0.7163 (2)	0.4998 (2)	0.0265 (5)	
C12	0.7040 (2)	0.7901 (2)	0.4412 (2)	0.0240 (5)	
C13	0.7758 (2)	0.8777 (2)	0.3541 (2)	0.0245 (5)	
C14	0.6902 (3)	1.0219 (2)	0.3089 (2)	0.0310 (5)	
H14	0.5843	1.0632	0.3339	0.037*	
C15	0.7568 (3)	1.1061 (2)	0.2284 (2)	0.0355 (6)	
H15	0.6975	1.2049	0.1987	0.043*	
C16	0.9113 (3)	1.0456 (3)	0.1907 (2)	0.0362 (6)	
H16	0.9579	1.1031	0.1350	0.043*	
C17	0.9966 (3)	0.9026 (3)	0.2340 (2)	0.0332 (5)	
H17	1.1020	0.8618	0.2076	0.040*	
C18	0.9314 (2)	0.8173 (2)	0.3153 (2)	0.0280 (5)	
H18	0.9914	0.7185	0.3448	0.034*	
C19	0.8432 (2)	0.7532 (2)	0.7740 (2)	0.0233 (5)	
C20	0.7332 (2)	0.8915 (2)	0.7556 (2)	0.0296 (5)	
H20	0.6946	0.9429	0.6700	0.036*	
C21	0.6802 (3)	0.9539 (3)	0.8632 (3)	0.0376 (6)	
H21	0.6040	1.0485	0.8519	0.045*	
C22	0.7381 (3)	0.8787 (3)	0.9879 (2)	0.0369 (6)	
H22	0.7005	0.9218	1.0617	0.044*	
C23	0.8501 (3)	0.7414 (3)	1.0049 (2)	0.0344 (5)	
H23	0.8901	0.6904	1.0901	0.041*	
C24	0.9038 (2)	0.6785 (2)	0.8972 (2)	0.0286 (5)	
H24	0.9817	0.5846	0.9080	0.034*	

### Atomic displacement parameters (Å^2^)

**Table:**

	*U*^11^	*U*^22^	*U*^33^	*U*^12^	*U*^13^	*U*^23^
S	0.0174 (2)	0.0248 (3)	0.0242 (3)	−0.0081 (2)	−0.00033 (18)	−0.0022 (2)
O1	0.0238 (7)	0.0305 (9)	0.0299 (9)	−0.0084 (6)	−0.0053 (6)	−0.0044 (7)
O2	0.0223 (7)	0.0265 (9)	0.0369 (10)	−0.0036 (6)	−0.0003 (6)	−0.0050 (7)
C1	0.0188 (9)	0.0223 (11)	0.0238 (11)	−0.0064 (8)	0.0017 (8)	−0.0074 (9)
C2	0.0193 (9)	0.0216 (11)	0.0274 (12)	−0.0063 (8)	0.0018 (8)	−0.0095 (9)
C3	0.0232 (10)	0.0224 (11)	0.0308 (12)	−0.0091 (8)	0.0083 (8)	−0.0112 (9)
C4	0.0261 (11)	0.0277 (12)	0.0332 (13)	−0.0113 (9)	0.0050 (9)	−0.0062 (10)
C5	0.0389 (12)	0.0335 (14)	0.0367 (14)	−0.0142 (10)	0.0070 (10)	−0.0046 (11)
C6	0.0474 (14)	0.0377 (15)	0.0431 (16)	−0.0235 (12)	0.0206 (12)	−0.0106 (12)
C7	0.0319 (12)	0.0411 (15)	0.0481 (16)	−0.0229 (11)	0.0184 (11)	−0.0193 (12)
C8	0.0244 (10)	0.0321 (13)	0.0389 (14)	−0.0115 (9)	0.0105 (9)	−0.0172 (11)
C9	0.0197 (10)	0.0421 (15)	0.0493 (16)	−0.0135 (10)	0.0068 (10)	−0.0207 (12)
C10	0.0211 (10)	0.0397 (14)	0.0417 (15)	−0.0075 (10)	−0.0035 (9)	−0.0158 (11)
C11	0.0225 (10)	0.0256 (12)	0.0316 (13)	−0.0071 (9)	0.0020 (8)	−0.0104 (9)
C12	0.0207 (9)	0.0244 (11)	0.0257 (12)	−0.0053 (8)	−0.0019 (8)	−0.0085 (9)
C13	0.0281 (10)	0.0268 (12)	0.0196 (11)	−0.0105 (9)	−0.0003 (8)	−0.0064 (9)
C14	0.0325 (11)	0.0265 (13)	0.0302 (13)	−0.0064 (9)	0.0002 (9)	−0.0072 (10)
C15	0.0466 (13)	0.0213 (12)	0.0367 (14)	−0.0115 (10)	−0.0019 (10)	−0.0036 (10)
C16	0.0468 (13)	0.0354 (14)	0.0331 (14)	−0.0249 (11)	0.0010 (10)	−0.0027 (11)
C17	0.0305 (11)	0.0376 (14)	0.0325 (13)	−0.0144 (10)	0.0019 (9)	−0.0064 (11)
C18	0.0290 (11)	0.0247 (12)	0.0282 (12)	−0.0075 (9)	−0.0010 (9)	−0.0051 (9)
C19	0.0208 (10)	0.0268 (12)	0.0257 (12)	−0.0128 (9)	0.0000 (8)	−0.0045 (9)
C20	0.0261 (10)	0.0292 (13)	0.0314 (13)	−0.0080 (9)	−0.0063 (9)	−0.0045 (10)
C21	0.0298 (12)	0.0363 (14)	0.0453 (16)	−0.0060 (10)	−0.0018 (10)	−0.0173 (12)
C22	0.0374 (13)	0.0480 (16)	0.0337 (14)	−0.0202 (11)	0.0059 (10)	−0.0199 (12)
C23	0.0453 (13)	0.0397 (14)	0.0251 (13)	−0.0252 (11)	−0.0016 (10)	−0.0027 (10)
C24	0.0335 (11)	0.0244 (12)	0.0288 (13)	−0.0137 (9)	−0.0030 (9)	−0.0009 (9)

### Geometric parameters (Å, °)

**Table:**

S—O2	1.4926 (15)	C12—C13	1.464 (3)
S—C1	1.7690 (19)	C13—C14	1.388 (3)
S—C19	1.801 (2)	C13—C18	1.402 (3)
O1—C12	1.376 (2)	C14—C15	1.377 (3)
O1—C11	1.380 (3)	C14—H14	0.9500
C1—C12	1.356 (3)	C15—C16	1.391 (3)
C1—C2	1.455 (3)	C15—H15	0.9500
C2—C11	1.373 (3)	C16—C17	1.375 (3)
C2—C3	1.426 (3)	C16—H16	0.9500
C3—C4	1.407 (3)	C17—C18	1.381 (3)
C3—C8	1.431 (3)	C17—H17	0.9500
C4—C5	1.369 (3)	C18—H18	0.9500
C4—H4	0.9500	C19—C24	1.380 (3)
C5—C6	1.406 (3)	C19—C20	1.383 (3)
C5—H5	0.9500	C20—C21	1.381 (3)
C6—C7	1.357 (4)	C20—H20	0.9500
C6—H6	0.9500	C21—C22	1.389 (4)
C7—C8	1.414 (3)	C21—H21	0.9500
C7—H7	0.9500	C22—C23	1.382 (3)
C8—C9	1.423 (3)	C22—H22	0.9500
C9—C10	1.355 (4)	C23—C24	1.386 (3)
C9—H9	0.9500	C23—H23	0.9500
C10—C11	1.406 (3)	C24—H24	0.9500
C10—H10	0.9500		
			
O2—S—C1	110.64 (9)	C1—C12—C13	133.87 (18)
O2—S—C19	106.60 (9)	O1—C12—C13	115.97 (18)
C1—S—C19	98.59 (9)	C14—C13—C18	119.2 (2)
C12—O1—C11	106.29 (16)	C14—C13—C12	120.17 (18)
C12—C1—C2	107.88 (17)	C18—C13—C12	120.62 (19)
C12—C1—S	119.06 (15)	C15—C14—C13	120.8 (2)
C2—C1—S	132.80 (16)	C15—C14—H14	119.6
C11—C2—C3	119.15 (18)	C13—C14—H14	119.6
C11—C2—C1	104.07 (19)	C14—C15—C16	119.7 (2)
C3—C2—C1	136.75 (18)	C14—C15—H15	120.2
C4—C3—C2	124.79 (18)	C16—C15—H15	120.2
C4—C3—C8	118.6 (2)	C17—C16—C15	119.9 (2)
C2—C3—C8	116.6 (2)	C17—C16—H16	120.1
C5—C4—C3	120.9 (2)	C15—C16—H16	120.1
C5—C4—H4	119.5	C16—C17—C18	121.0 (2)
C3—C4—H4	119.5	C16—C17—H17	119.5
C4—C5—C6	120.6 (2)	C18—C17—H17	119.5
C4—C5—H5	119.7	C17—C18—C13	119.4 (2)
C6—C5—H5	119.7	C17—C18—H18	120.3
C7—C6—C5	119.8 (2)	C13—C18—H18	120.3
C7—C6—H6	120.1	C24—C19—C20	121.2 (2)
C5—C6—H6	120.1	C24—C19—S	118.22 (16)
C6—C7—C8	121.5 (2)	C20—C19—S	120.25 (17)
C6—C7—H7	119.2	C21—C20—C19	119.0 (2)
C8—C7—H7	119.2	C21—C20—H20	120.5
C7—C8—C9	121.0 (2)	C19—C20—H20	120.5
C7—C8—C3	118.5 (2)	C20—C21—C22	120.2 (2)
C9—C8—C3	120.5 (2)	C20—C21—H21	119.9
C10—C9—C8	122.5 (2)	C22—C21—H21	119.9
C10—C9—H9	118.8	C23—C22—C21	120.2 (2)
C8—C9—H9	118.8	C23—C22—H22	119.9
C9—C10—C11	116.1 (2)	C21—C22—H22	119.9
C9—C10—H10	122.0	C22—C23—C24	119.8 (2)
C11—C10—H10	122.0	C22—C23—H23	120.1
C2—C11—O1	111.62 (17)	C24—C23—H23	120.1
C2—C11—C10	125.1 (2)	C19—C24—C23	119.5 (2)
O1—C11—C10	123.3 (2)	C19—C24—H24	120.3
C1—C12—O1	110.10 (18)	C23—C24—H24	120.3
			
O2—S—C1—C12	−136.67 (16)	C9—C10—C11—C2	−0.1 (3)
C19—S—C1—C12	111.89 (17)	C9—C10—C11—O1	179.1 (2)
O2—S—C1—C2	50.0 (2)	C2—C1—C12—O1	1.4 (2)
C19—S—C1—C2	−61.5 (2)	S—C1—C12—O1	−173.45 (13)
C12—C1—C2—C11	−0.5 (2)	C2—C1—C12—C13	178.2 (2)
S—C1—C2—C11	173.40 (17)	S—C1—C12—C13	3.3 (3)
C12—C1—C2—C3	177.6 (2)	C11—O1—C12—C1	−1.8 (2)
S—C1—C2—C3	−8.5 (4)	C11—O1—C12—C13	−179.21 (17)
C11—C2—C3—C4	178.0 (2)	C1—C12—C13—C14	−129.7 (3)
C1—C2—C3—C4	0.1 (4)	O1—C12—C13—C14	47.0 (3)
C11—C2—C3—C8	−0.6 (3)	C1—C12—C13—C18	50.2 (3)
C1—C2—C3—C8	−178.5 (2)	O1—C12—C13—C18	−133.2 (2)
C2—C3—C4—C5	−179.5 (2)	C18—C13—C14—C15	−0.8 (3)
C8—C3—C4—C5	−0.9 (3)	C12—C13—C14—C15	179.1 (2)
C3—C4—C5—C6	0.0 (3)	C13—C14—C15—C16	0.6 (4)
C4—C5—C6—C7	0.9 (4)	C14—C15—C16—C17	−0.1 (4)
C5—C6—C7—C8	−0.8 (4)	C15—C16—C17—C18	−0.3 (4)
C6—C7—C8—C9	178.5 (2)	C16—C17—C18—C13	0.1 (3)
C6—C7—C8—C3	−0.2 (3)	C14—C13—C18—C17	0.5 (3)
C4—C3—C8—C7	1.1 (3)	C12—C13—C18—C17	−179.4 (2)
C2—C3—C8—C7	179.70 (19)	O2—S—C19—C24	16.08 (18)
C4—C3—C8—C9	−177.6 (2)	C1—S—C19—C24	130.73 (17)
C2—C3—C8—C9	1.0 (3)	O2—S—C19—C20	−170.04 (16)
C7—C8—C9—C10	−179.7 (2)	C1—S—C19—C20	−55.39 (18)
C3—C8—C9—C10	−1.1 (3)	C24—C19—C20—C21	−1.9 (3)
C8—C9—C10—C11	0.6 (3)	S—C19—C20—C21	−175.57 (17)
C3—C2—C11—O1	−179.15 (17)	C19—C20—C21—C22	0.6 (3)
C1—C2—C11—O1	−0.6 (2)	C20—C21—C22—C23	0.6 (4)
C3—C2—C11—C10	0.1 (3)	C21—C22—C23—C24	−0.5 (3)
C1—C2—C11—C10	178.7 (2)	C20—C19—C24—C23	2.0 (3)
C12—O1—C11—C2	1.5 (2)	S—C19—C24—C23	175.80 (15)
C12—O1—C11—C10	−177.8 (2)	C22—C23—C24—C19	−0.8 (3)

### Hydrogen-bond geometry (Å, °)

**Table:**

*D*—H···*A*	*D*—H	H···*A*	*D*···*A*	*D*—H···*A*
C9—H9···O2^i^	0.95	2.59	3.488 (3)	158
C18—H18···O2^ii^	0.95	2.57	3.323 (3)	137

Symmetry codes: (i) *x*−1, *y*, *z*; (ii) −*x*+2, −*y*+1, −*z*+1.

## References

[bb1] Brandenburg, K. (1998). *DIAMOND* Crystal Impact GbR, Bonn, Germany.

[bb2] Bruker (2001). *SAINT* and *SMART* Bruker AXS Inc., Madison, Wisconsin, USA.

[bb3] Choi, H. D., Seo, P. J., Son, B. W. & Lee, U. (2007). *Acta Cryst.* E**63**, o1731–o1732.

[bb4] Choi, H. D., Seo, P. J., Son, B. W. & Lee, U. (2008). *Acta Cryst.* E**64**, o727. 10.1107/S1600536808007046 PMC2961025 21202117

[bb5] Farrugia, L. J. (1997). *J. Appl. Cryst.* **30**, 565.

[bb6] Goel, A. & Dixit, M. (2004). *Tetrahedron Lett.* **45**, 8819-8821.

[bb7] Hagiwara, H., Sato, K., Suzuki, T. & Ando, M. (1999). *Heterocycles*, **51**, 497-500.

[bb8] Piloto, A. M., Costa, S. P. G. & Goncalves, M. S. T. (2005). *Tetrahedron Lett.* **46**, 4757–4760.

[bb9] Sheldrick, G. M. (2008). *Acta Cryst.* A**64**, 112–122. 10.1107/S0108767307043930 18156677

